# Stroboscopic visual training in basketball: a narrative review integrating cross-sport evidence and a hypothetical framework based on the information processing model

**DOI:** 10.3389/fpsyg.2026.1876582

**Published:** 2026-06-19

**Authors:** Hao Kang, Xiaofen Cao

**Affiliations:** School of Physical Education, Hunan Agricultural University, Changsha, Hunan, China

**Keywords:** basketball, information processing model, measurement indicators, sport performance, sports vision training, stroboscopic visual training

## Abstract

Given the scarcity of basketball-specific stroboscopic visual training (SVT) research and the lack of a systematic synthesis linking training parameters to distinct categories of improvement indicators, this review retrieved studies on SVT in open-skill sports comparable to basketball published between 1997 and 2026. Through a narrative review, we analyzed cross-sport evidence relevant to any stage of the information processing model to examine the relationship between SVT parameters and post-training improvement indicators. Based on this synthesis, we propose a three-phase parameter framework for basketball, integrating basketball-specific measures to construct a hypothetical parameter–effect matching framework for SVT in basketball based on the information processing model. The aim is to summarize and analyze which SVT parameters can influence neurophysiological or cognitive-psychological indicators of athletic performance, map these influences onto stages of the information processing model, and develop a hypothesis framework to guide applications of SVT in basketball. The review found that SVT can improve three indicators associated with the information processing model: reaction time, decision-making accuracy, and motor control. However, the parameter recommendations for each phase of this framework represent trends derived from existing cross-sport research, and the specific parameter values await further validation in future basketball-specific studies.

## 1 Introduction

In current competitive sport, athletic performance depends on the coordination of the nervous and perceptual systems. For open, adversarial sports such as basketball, the outcome is related to an athlete’s ability to perceive, decide, and execute actions in response to dynamically changing visual information (Lochhead et al., 2024).

However, traditional basketball training has focused on physical, technical, and tactical dimensions, with systematic training of sport-specific visual-cognitive abilities receiving less attention, and a lack of varied training methods and quantitative assessment. Prolonged reliance on traditional basketball-specific training alone may not address the need for improved visual-cognitive abilities. It can leave athletes less prepared for the adaptation, regulation, and decision-making demands that arise under visually restricted conditions. This, in turn, may lead to reliance on prior experience during competition and limit the expression of their athletic potential.

Against this background, training techniques designed to challenge and enhance visual information processing capacity have received attention, and SVT is one such approach. SVT employs intermittent visual occlusion to impose visual-cognitive load, requiring individuals to utilize the limited visual input they receive. This process aims to increase visual sensitivity and improve visual skills when normal visual conditions are restored (Appelbaum and Erickson, 2018). Historically, American basketball player Michael Jordan is considered one of the earliest athletes to have incorporated SVT into his training regimen, suggesting its potential for high-level athletes and indicating the importance of vision-oriented training (Haberstroh, 2016). Enhancement of young basketball players’ game performance also requires the training of visual-perceptual abilities, enabling them to deliver more assists and perform more efficiently (Zemková et al., 2025). In recent years, with the commercial availability of wearable stroboscopic devices (e.g., Senaptec Strobe, Nike), SVT has received attention in scientific sports training, and exploratory studies across various sports have increased.

Although several SVT research cases now exist, shifting the perspective to basketball reveals two gaps that constrain application and development. First, empirical research is insufficient in both quantity and focus. To date, SVT studies involving basketball players are limited, with two studies identified—one of which is a randomized controlled trial mixing basketball and soccer athletes (Appelbaum et al., 2012; Li et al., 2026). Investigations targeting practical competitive performance indicators, such as reduced reaction time and landing control, are lacking. Second, training application lacks scientific guidance, and the scope of SVT effects remains undefined, which may limit coaches’ motivation to integrate SVT into basketball training design. The relationship between key SVT parameters (e.g., frequency, duty cycle) and basketball-specific performance indicators (e.g., reaction time, shooting accuracy) has not been systematically delineated, and the logic for parameter configuration remains unclear. During the literature review, we noted that the indicators improved by different SVT parameters could be mapped, to a certain degree, onto the three stages of the information processing model. Given that open-skill sports share similar cognitive demands and that SVT produces improvements in reactive abilities in these athletes compared to those from closed-skill sports (Heilmann et al., 2022; Wang et al., 2025), this study integrated SVT research from open-skill sports to construct a hypothetical parameter–effect theoretical framework grounded in the information processing model. This work is intended to provide a hypothesis system and a prototype training protocol for future empirical SVT research in basketball, thereby advancing sports vision training theory and promoting sport-specific training practices.

Cross-sport evidence is employed because basketball shares strong visual-cognitive demands with the included open-skill sports. The fast game pace, dense multi-player interactions, reliance on peripheral vision and body cues, and the need for rapid decision-making under time pressure and incomplete visual information distinguish basketball from closed-skill sports and align it closely with the perceptual–cognitive demands targeted by SVT. These features make basketball a particularly relevant context for SVT application and justify extracting parameter–effect evidence from other open-skill sports to construct a hypothetical framework for basketball.

## 2 Methods

Given the limited number of basketball-specific SVT studies and the heterogeneity among existing cross-sport studies, not all of which involve open-skill sports, a narrative review approach was adopted. This method permits the integration of diverse evidence types from different sports and facilitates the construction of a hypothesis framework that can serve as a reference for future basketball-specific SVT research. The primary objective of this review is to construct a hypothetical parameter–effect matching framework for stroboscopic visual training (SVT) in basketball, grounded in the information processing model. Three secondary objectives are: To analyze existing SVT evidence concerning effects on perceptual–motor processing at the stages of stimulus identification, response selection, and response programming; To map key SVT parameters (frequency, duty cycle, intervention duration, weekly frequency, and session length) onto behavioral and neural indicators corresponding to the three stages (reaction time, decision-making accuracy, motor control); And to identify the principal training-related variables that may shape performance outcomes, thereby providing a hypothesis prototype for future basketball-specific empirical SVT research. A literature search was conducted in PubMed, Web of Science, and EBSCO from November 19, 2025, to April 17, 2026, using specific key words such as “Strobe, ““stroboscopic visual training,” “strobe glasses,” “reaction time,” “decision-making,” “basketball,” and “athletic performance.”

### 2.1 Inclusion and exclusion criteria

A search was conducted based on titles and abstracts. The following inclusion criteria were applied: 1. studies focusing on SVT in sport and its effects on athletic performance task indicators that can be linked to specific stages of the information processing model; 2. studies published between 1997 and 2026 and indexed in Web of Science, PubMed, or EBSCO; 3. studies that specifically addressed SVT parameters, athletic performance indicators, the relationships between these indicators and different parameter configurations, and the consequent effects on sport performance. The following exclusion criteria were applied: 1. studies that did not address athletic performance indicators; 2. studies in which SVT was combined with additional forms of sensory stimulation, such as auditory cues, medical education, or virtual reality; 3. articles not published in peer-reviewed journals; and 4. publications lacking a scientific or methodical approach, such as opinion pieces and editorials. Priority was given to studies and systematic reviews concerned with SVT parameter settings and sport performance enhancement. Attention was directed toward studies offering insight into the neural mechanisms underlying SVT, so as to select work relevant to the central objective of this review.

### 2.2 Construction of the hypothesis framework

The hypothesis framework was constructed using the classic information processing model in conjunction with the motor performance process (Schmidt et al., 2018). This hypothesis framework was formed by aggregating the indicators—across SVT studies in open-skill sports similar to basketball and some closed-skill sports—that correspond to improvements from different SVT parameters, along with the associated sport performance enhancements, thereby establishing a correspondence between parameters and effects. Finally, the interaction among information processing stages in sport, different stroboscopic parameters, and various indicators and sport performance outcomes was integrated to create the hypothesis framework.

### 2.3 Derivation logic for the hypothesis framework

The information processing model divides perceptual-motor processing into three stages—stimulus identification, response selection, and response programming—providing a theoretical basis for understanding how visual information is processed during sport. By intermittently interrupting visual input, SVT compresses the quality and available acquisition time of information and is therefore likely to have an impact on the stimulus identification stage, with effects on the subsequent stages. Based on this logic, it can be inferred that the effects of SVT on each stage of the information processing model can be assessed through behavioral indicators. Changes in stimulus identification efficiency are reflected in reaction time; changes in response selection efficiency are related to decision-making accuracy; and changes in response programming efficiency are manifested in indicators such as movement stability and balance. In published SVT studies, the improved indicators align with these expectations, such as shortened reaction time (Wang et al., 2025), improved decision-making accuracy (Li et al., 2026), and enhanced balance ability (Lu et al., 2025). Accordingly, the information processing model was adopted as the theoretical foundation for the hypothesis framework.

## 3 Theoretical background: stages of information processing in sport

### 3.1 Stimulus identification stage

The function of the stimulus identification stage is to detect and recognize stimuli present in the environment. This stage typically occurs first in the information processing sequence of motor performance. Individuals actively or passively use the visual system and the ventral pathway to search for and capture various stimuli from the surroundings, filtering them to select those with potential relevance to the ongoing activity. Research indicates that elite athletes possess anticipatory capabilities within their motor system, enabling them to initiate motor program preparation before visual input arrives (Aglioti et al., 2008). Through its provision of incomplete, intermittent visual information, SVT may further strengthen this anticipation-based perception-action coupling mechanism, potentially providing a temporal advantage at the stimulus identification stage. An increase in reaction time may be associated with a prolonged stimulus identification stage (Czyż, 2021). Rapid and accurate identification of relevant stimuli may help athletes in competition enter the response selection stage earlier, offering an advantage. SVT has been shown to shorten athletes’ reaction time to some degree, which is consistent with the demands of this stage (Jothi et al., 2025). Therefore, the goal for this stage is to enhance stimulus identification speed and reduce the reaction time required to recognize visual stimuli. In basketball, reaction time can be reflected by indicators such as defensive motor response time and steal motor response time.

### 3.2 Response selection stage

The response selection stage involves making a response decision tailored to the identified stimulus pattern (Czyż, 2021). After stimulus identification in the first stage, the acquired information is analyzed in conjunction with the real-world context, with the goal of making an optimal decision. This stage serves as the intermediary linking information input to motor control and is an important part of the motor performance process. Intermittently restricting visual information input reduces the availability of visual information to some extent. Under these conditions, the motor-cognitive system shifts from automatic control to attentional control, thereby improving the encoding of visual information and the retention of transient cues in short-term memory (Appelbaum et al., 2011). A recent systematic review on SVT discussed the training effect on this stage. While the review noted that there is currently no conclusive evidence that training under intermittent visual occlusion can directly enhance athletes’ decision-making capacity (Wang et al., 2025), several considerations warrant its inclusion. In multiple studies, decision-making improvement has been used as an observation dimension for sport performance enhancement following SVT intervention, given its role in the complete motor performance process. Furthermore, the ability to acquire functional information from movement, which is indirectly associated with stage one, may contribute to decision-making improvement (Michaels, 2000; Mann et al., 2010). Additionally, one recent basketball-specific SVT study found that basketball players’ decision-making ability improved after a sport-specific training intervention incorporating SVT (Li et al., 2026). We therefore decided to include the response selection stage in this review’s discussion and the construction of the hypothesis framework, exploring the potential effects of SVT on this stage. Consequently, the parameter settings for this stage are more exploratory than those for the other two stages and require verification through future SVT studies. In basketball, indicators such as assist-to-turnover ratio and decision-making accuracy can reflect whether this stage is affected or improved through SVT.

### 3.3 Response programming stage

As the final stage of the information processing model, the individual must translate the idea formed in the response selection stage into specific, implementable commands for the motor system (Czyż, 2021). Because this stage occurs prior to movement execution, the internal programming of motor commands within the brain cannot be directly observed. Although few experiments have directly targeted response programming, a number of studies have indirectly inferred its optimization by measuring improvements in motor control. For instance, Kim et al. (2017) investigated the value of SVT concerning visual feedback dependency for postural control in patients with somatosensory deficits, contributing to sensory reorientation within multisensory integration. Compared to balance training alone, incorporating SVT into balance training led to improvements in perceived ankle instability and dynamic postural control, with benefits for athletic patients (Kim et al., 2021). SVT may alter the degree of visual dependency in athletes with chronic ankle instability, and it can help reduce visual input and enhance motor control during specific activity phases in the rehabilitation process (Uzlaşır et al., 2021; Demir and Bilgin, 2025; Luo et al., 2025b). Regarding landing control after anterior cruciate ligament reconstruction, the assistive rehabilitation function of SVT has shown a role in altering the risk of Anterior Cruciate Ligament (ACL) injury, and it has been noted that the intermittent visual input training provided by SVT offers a simple, easy-to-implement, and novel stressor for the neural control system (Grooms et al., 2015; Grooms et al., 2018). SVT can alter visual reliance during static and dynamic postural control, enhancing movement stability (Lee et al., 2022a). In a study involving healthy female athletes, wearing stroboscopic goggles during virtual neural training reduced landing errors and decreased connectivity between the left somatosensory motor cortex and the left frontal pole and left postcentral gyrus, suggesting that SVT may have a role in reducing the cognitive and sensory dependence of knee motor control (Wohl et al., 2024). SVT has an impact on several different visual and motor performance outcomes, including balance and hand-eye coordination (Das et al., 2023; Lu et al., 2025). Results from a basketball SVT study indicate that SVT can improve coordination in basketball players (Li et al., 2026). Foundational motor learning research shows that real-time visual feedback can partially inhibit the improvement of feedforward control, whereas reducing online visual feedback promotes faster and more automatic movement performance, providing analogical evidence for SVT’s potential to enhance this stage (Raichin et al., 2021). It should be clarified here that much of the above evidence originates from the rehabilitation domain, and that response programming and motor control are related but distinct concepts. Similarly, while SVT research cannot directly observe improvements in response programming, the improvements in motor control observed across the studies mentioned above may reflect optimization in response programming. Therefore, this review treats motor control as an indirect assessment of changes in the response programming stage, corresponding in basketball to indicators such as shooting form, landing control, and movement stability.

## 4 Positioning of SVT and basketball-specific visual-cognitive demands

### 4.1 Comparison of SVT with traditional visual training methods

To clarify the value and positioning of SVT within the basketball training system, it is necessary to systematically compare it with two established sport-specific visual-cognitive training methods: quiet eye training and multiple object tracking training. The main difference in the intervention logic among the three lies in SVT’s principle of deprivation and adaptation. By intermittently and rhythmically interrupting visual input, SVT creates controllable perceptual difficulties, requiring the nervous system to improve predictive and adaptive capabilities under conditions of incomplete information and reduce reliance on visual feedback, thereby improving information processing efficiency when normal vision is restored (Das et al., 2023). Quiet eye training aims to improve performance; by extending the duration of gaze fixation on a relevant target before the execution of a key movement, it improves fixation stability, enhances attentional focus, reduces interference, stabilizes motor programs, accelerates skill acquisition, and helps reduce anxiety in athletes (Moore et al., 2012; Moeinirad et al., 2022). Multiple object tracking training aims at attention allocation and information filtering, primarily challenging the breadth and distribution of dynamic visual attention, as well as the ability to continuously track and filter targets under interference, ultimately serving to enhance decision-making accuracy (Mangine et al., 2014; Martín et al., 2017).

The three methods impose load on different dimensions. SVT can impose perceptual load while preserving elements of ecological validity (Ren et al., 2025). This includes temporal pressure and working memory load, challenging the brain’s ability to predict the temporal structure of the visual information stream and to integrate and maintain fragmented information online. Quiet eye training imposes attentional selection and stability load (Vine and Wilson, 2011). Multiple object tracking training imposes spatial attention allocation and working memory updating load (Ehmann et al., 2021).

Based on these distinctions, each method has its own application scenarios. SVT, when combined with actual court settings, may be applied to open, fast-paced decision-making scenarios in basketball, such as defensive anticipation during fast breaks, timing for penetrating passes, and improving the processing efficiency of underlying visuomotor neural pathways. Quiet eye training has empirical support for execution stability under pressure in closed, high-precision tasks with relatively ample preparation time, such as free throws and spot-up jump shots (Vickers et al., 2019). Multiple object tracking training is suited for enhancing an athlete’s situational awareness and decision-making ability, particularly for reading complex offensive formations, spotting open teammates on the court, and tracking the dynamics of off-ball players (Romeas et al., 2016).

SVT is not intended to replace traditional basketball technical/tactical training or other cognitive training but rather to occupy a bridging ecological niche for transferring training effects from the laboratory to the training ground. As a neural efficiency enhancer, SVT neither directly incubates new techniques nor isolates the training of a specific cognitive function. By embedding visual perturbation within sport-specific tasks, SVT can target the brain’s speed and adaptability in processing visuomotor information, and it can be viewed as a bridge connecting traditional physical or technical training with sports intelligence.

### 4.2 Specificity of visual-cognitive demands in basketball

Among various sports, basketball presents specific demands on the visual-cognitive system. As an open-skill, fast-paced team sport, its specific demands can be outlined in three main aspects based on existing research: the information processing time window, the preferential reliance on and partial inhibition of body cues, and the neural basis of perception-action coupling.

Basketball players are required to process visual information rapidly within a dynamic competitive environment. Athletes with stronger perceptual-cognitive abilities may make faster decisions because they can assess a given scenario more quickly, thus gaining extra motor response time (Mangine et al., 2014). Even within the same sport of basketball, when simultaneously processing the dynamic information of more than five players, high-level players exhibit faster average reaction times than novices. Under conditions where central or peripheral vision is partially impaired, the information processing advantage of high-level basketball players persists when full vision is available (Ryu et al., 2015; Jin et al., 2023; Yu et al., 2025). This indicates that basketball playing level is correlated with reaction time. The brief processing time and incomplete visual field require basketball players to make anticipatory judgments and decisions within a short cognitive window, rather than waiting for complete visual input before programming and executing an action. The compressed information processing time in competition bears partial similarity to the intermittent visual interruption environment created by SVT. Considering the wearability of SVT devices and the transferability of their effects to competition, improving the efficiency of stimulus identification and information processing through SVT can be important for basketball performance.

Unlike sports that rely more on the trajectory of a ball, the physically confrontational nature of basketball means that players tend to rely more on processing body-related information when anticipating an opponent’s actions or a shot outcome (Aglioti et al., 2008; Wu et al., 2013; Uchida et al., 2014; Li et al., 2023). Furthermore, when deceptive actions (e.g., head fakes) occur, basketball players need to discriminate against such body information and adapt after experiencing the resulting response conflict, inhibiting the processing of interfering information (Kunde and Wühr, 2006). High-level basketball players also demonstrate higher accuracy in anticipating deceptive actions compared to novices (Alemanno et al., 2025). In that study, only basketball experts exhibited this adaptation, while soccer experts and non-athletes did not. Moreover, the effect of deception was reduced under cognitive load intervention (Weigelt et al., 2017; Güldenpenning et al., 2020). The extraction of body information and adaptation to deception serve, to a certain degree, as features distinguishing basketball from other team-based adversarial sports, clarifying the specific needs and direction for sport-specific training.

During gameplay, players also continuously observe their own and others’ movements to adjust movement quality or shooting accuracy. Research has found that when elite basketball players observe an erroneous action, their corticospinal excitability is higher than when observing a correct action (Aglioti et al., 2008). This indicates that the corticospinal system provides the neural substrate for the visual-cognitive demand of capturing and correcting erroneous movements. By intermittently disrupting vision, SVT can increase this visual-cognitive demand, potentially enabling the corticospinal system to translate prefrontal anticipatory activity into motor output under conditions of restricted visual input.

In summary, the three specific visual-cognitive demands of basketball define the potential application goals of SVT in this sport: improving early information extraction efficiency, inhibiting irrelevant interference, and enhancing prefrontal anticipatory activity through intermittent visual disruption.

## 5 Classification of indicators in SVT training effects

### 5.1 Temporal indicators

Across existing meta-analyses, the effect-size estimates for SVT on reaction time appear relatively consistent: Wang et al. (2025) reported a medium-to-large effect, Luo et al. (2025a) also observed significant improvements on time-related performance indicators, and Huelsduenker et al. (2021b) reported significant short- and long-term improvements in elite youth athletes. This convergence indicates that reaction time is among the most robust dimensions on which SVT exerts an improvement effect. A volleyball SVT study focusing on agility performance also found an improvement in the reaction index after training, with an interaction between group and time (Zwierko et al., 2024a). Furthermore, research has suggested that this reduction in reaction time may stem from an SVT-enhanced ability to anticipate action timing (Smith and Mitroff, 2012). Cross-sport evidence indicates an effect of SVT on temporal indicators, providing a reference for the potential use of measuring indicators such as defensive motor response time and drive initiation motor response time in basketball. However, since current evidence largely originates from laboratory or closed-skill tasks, the transfer of ecological validity to basketball’s open competitive context still requires verification.

### 5.2 Accuracy indicators

An SVT study involving mixed athletes from Duke University’s Ultimate Frisbee and American football teams showed an improvement in accuracy on a central attention task (Appelbaum et al., 2011). A subsequent SVT study also reported an increase in accuracy after training (Mitroff et al., 2013). Defensive accuracy in badminton could also be improved through SVT (Hülsdünker et al., 2019). Concerning accuracy indicators, however, three meta-analyses have yielded different results. One meta-analysis showed a moderate effect of SVT on accuracy outcomes (Luo et al., 2025a), and another suggested that improvements in hand-eye coordination precision could be obtained through SVT (Ren et al., 2025). Yet, a third meta-analysis found that compared to the improvement in temporal indicators, the overall effect of SVT on task accuracy indicators did not reach statistical significance (Wang et al., 2025). Taken together, effect-size estimates for accuracy outcomes across meta-analyses show clear heterogeneity, ranging from medium significant effects (Luo et al., 2025a; Ren et al., 2025) to non-significant effects (Wang et al., 2025). This variability suggests that the impact of SVT on accuracy is substantially moderated by task type, parameter configuration, and sport-specific characteristics, a finding that itself motivates the construction of a stage and parameter-specific hypothetical framework. This suggests that the effects of SVT may not enhance all performance dimensions across different sports but are rather characterized by specificity. When using precision indicators such as shooting percentage or passing success rate as criterion measures in basketball, it is necessary to consider their matching relationship with specific training parameters, rather than evaluating the overall effect of SVT generically.

### 5.3 Neurophysiological indicators

Regarding improvements in response programming, athletes may achieve more efficient motor execution by economizing saccadic processing and integrating visual information, thereby optimizing sensory-cognitive resource allocation and enhancing individual coordination (Zwierko et al., 2023). Foundational neuroscience research suggests that dorsal stream activity is associated with motor planning, and its downstream corticospinal motor system possesses a refined perceptual modulation capacity, capable of detecting erroneous or ineffective body configurations at an early stage (Aglioti et al., 2008). In other words, under conditions of restricted visual input created by SVT, athletes are forced to increase reliance on proprioception and feedforward control. Once an error in movement occurs, the athlete’s corticospinal system exhibits higher excitability compared to when a correct movement is produced, facilitating error recognition and correction. Long-term training may further modulate the sensitivity of the corticospinal system, allowing for the maintenance of movement stability even under perturbation. As for shortening reaction time, one reason may be the neurophysiological mechanisms activated by SVT (Wang et al., 2025). Research indicates that SVT can enhance central visual sensitivity and information processing efficiency (Appelbaum et al., 2011). Following SVT intervention, the conduction of visual signals from the retina to the primary cortex was accelerated; the latency of the P100 component of visual evoked potentials in handball players was shortened, particularly in the peripheral visual field, reflecting improved early visual processing (Zwierko et al., 2024b). In studies on badminton players, researchers observed a negative correlation between the latency of the N2 component in event-related potentials and visuomotor perception speed, as well as a positive effect of SVT on shortening N2 latency (Huelsduenker et al., 2021a,b). SVT shortens the N2 visual evoked potential latency to accelerate early visual processing and prolongs the N2-r potential latency to improve the efficiency of converting visual information into motor signals; the combined optimization reduces the overall visuomotor latency, shortening reaction time (Ren et al., 2025). This provides indirect evidence linking low-level neural adaptation to mid-level behavioral performance. Similarly, research on cognitive-motor dual-task training—a paradigm distinct from SVT logic but likewise involving the imposition of cognitive load during movement—offers indirect insight into SVT’s attentional control mechanisms. Semi-elite basketball players exhibited enhanced prefrontal negativity during a cognitive task, manifested as an earlier onset latency and larger amplitude (Lucia et al., 2021). These neural changes were associated with a decrease in error rate, suggesting that such training strengthens athletes’ active inhibition and top-down attentional capacity.

## 6 Matching SVT parameters with the three stages of the information processing model

It should be noted at the outset that the mappings between SVT parameters and stage-specific neural/behavioural indicators outlined below are trend-based syntheses drawn from cross-sport evidence. They are hypothetical propositions within the present framework rather than established causal relations, and are intended to guide—and to be tested by—future basketball-specific empirical research. The use of hedged wording (“may,” “suggests,” “is consistent with”) in describing parameter–indicator relationships throughout this section reflects this hypothetical stance.

### 6.1 Core visual parameters: stage-specific settings for frequency and duty cycle

Subgroup analyses from meta-analyses provide preliminary direction regarding two core visual parameters. For frequency, lower frequency settings (<10 Hz) were associated with a greater reduction in reaction time, consistent with the stage one setting of the present hypothesis framework, suggesting that low frequency may contribute to speed improvement by improving temporal anticipation (Wang et al., 2025). For duty cycle, settings in the low-to-moderate range (typically ≤50%) showed more benefit (Wang et al., 2025). An SVT intervention using low frequency (3–5 Hz) and a moderate level (Level 2–4) demonstrated benefits for transient attention in central vision, with gains reflected in speed improvement (Appelbaum et al., 2011). Another study indicated that a combination of low-to-moderate frequency and a low-to-moderate duty cycle had an effect on improving speed control, targeting the neuroplastic adaptation of the visual and multisensory systems under conditions of restricted visual field (Li et al., 2024). These two pieces of evidence suggest that visual interruptions at a low-to-moderate frequency and low-to-moderate duty cycle may impose more predictive demand on the trainee than intense and rapid visual deprivation, without causing a complete loss of the visual field, and can stimulate speed improvement in the dorsal stream, thereby improving reaction speed. This aligns with the intervention mechanism of SVT, which promotes neural adaptation through progressive difficulty increases, and provides a basis for the parameter configuration of stage one.

When the difficulty was increased from a low-to-moderate frequency and higher duty cycle (4 Hz, 6 Hz; 60%) to a low frequency and high duty cycle (1.33 Hz; 86.7%), the information processing efficiency of high-level athletes declined, with accuracy decreasing compared to average-level athletes. The prolonged visual occlusion resulted in a shift from automatic control to attentional control but improved the ability to retain visual information in short-term memory (Appelbaum et al., 2012; Beavan et al., 2021). Therefore, moderate frequency or low-to-moderate frequency with a higher duty cycle may be applicable in adaptive training, reducing the precision of sensory input to influence athletes’ processing decisions in cognitive and perceptual tasks (Harris et al., 2025). Moreover, a basketball-specific study indicated that SVT settings involving a medium-high frequency and higher duty cycle (9 Hz, 15 Hz; 50–70%) could improve the decision-making ability of collegiate basketball players, providing reference parameters for stage-two training that targets decision-making in conditions approximating actual competition (Li et al., 2026).

Furthermore, SVT at low frequencies (2.25 Hz, 4 Hz) with high duty cycles (50, 77.5%) combined with balance training was associated with a reduction in visual dependency during static and dynamic postural control in healthy individuals. Under visually restricted conditions, participants adjusted the sensory weighting of visual input to compensate for reduced somatosensory information (Lee et al., 2022a). Two studies on improving postural control in patients with chronic ankle instability and one study on enhancing balance ability in male collegiate soccer players all employed a low-frequency, high-duty-cycle parameter setting (3 Hz; 70%) (Han et al., 2022; Lee et al., 2022b; Lu et al., 2025). SVT under a low-to-moderate frequency and high duty cycle (1.33 Hz, 4 Hz; 60, 86.7%) constrained the dribbling speed of soccer players, with a decline observed at higher skill levels. The results suggested that SVT might be a useful auxiliary training tool for reducing dependence on incoming visual information during complex motor skills (Fransen et al., 2017). The same parameters also improved hand-eye coordination (Ellison et al., 2020). Two further pieces of evidence, both employing a low-frequency, high-duty-cycle setting, indicated that SVT could alter the mechanics of depth jumps, increasing jump intensity (1.75 Hz, 4 Hz; 60, 82.5%) and showing potential to reduce injury risk beyond traditional measures (2.86 Hz, 5 Hz; 50, 71.4%) (Grooms et al., 2018; Kroll et al., 2023). This body of evidence suggests an influence of a higher duty cycle on in-motion body adjustments and provides case-based evidence to support the construction of the low-frequency, high-duty-cycle parameter setting for the motor control component of stage three in the present hypothesis framework.

### 6.2 Training dose parameters: stage-specific settings for duration, frequency, and session length

In addition to visual parameters, the optimization of training dose parameters is also important. Based on an induction of existing evidence, relatively short-term (1–6 weeks), low-frequency (1–2 sessions per week), and short-duration (approximately 10 min per session) protocols improve reaction time (Wang et al., 2025). If the goal is to enhance overall sport performance, the duration, frequency, and session length need to be proportionally extended (Luo et al., 2025a). A basketball-specific SVT study demonstrated that increasing the number of weeks and session frequency (8 weeks, 3 sessions per week) while extending the session duration (approximately 40 min) could be suitable for improving decision-making ability in basketball players (Li et al., 2026). However, the value ranges for these dose parameters vary across studies. A 12-week SVT combined with balance training program enhanced static and dynamic balance ability in male collegiate soccer players—a sport that shares some cognitive demands with basketball and also requires physical confrontation (Lu et al., 2025). Studies on improving postural control and reducing visual dependency in chronic ankle instability patients suggest a minimum effective dose threshold for training duration (4 weeks), which must be met for improvements to occur (Lee et al., 2022b).

### 6.3 Research gaps and prospects regarding dose–response relationships

In summary, current SVT research exhibits an incomplete system in terms of parameter configuration. While there is a preliminary understanding of the effective ranges of individual core parameters, further exploration is still needed regarding multi-parameter combinations, the dose–response relationship, and how these parameters differentially affect the various information processing stages. This represents the core challenge that the present review’s proposed hypothetical parameter–effect theoretical framework aims to address.

## 7 Construction of a hypothetical parameter–effect matching framework for stroboscopic visual training in basketball based on the information processing model

**Figure 1:**
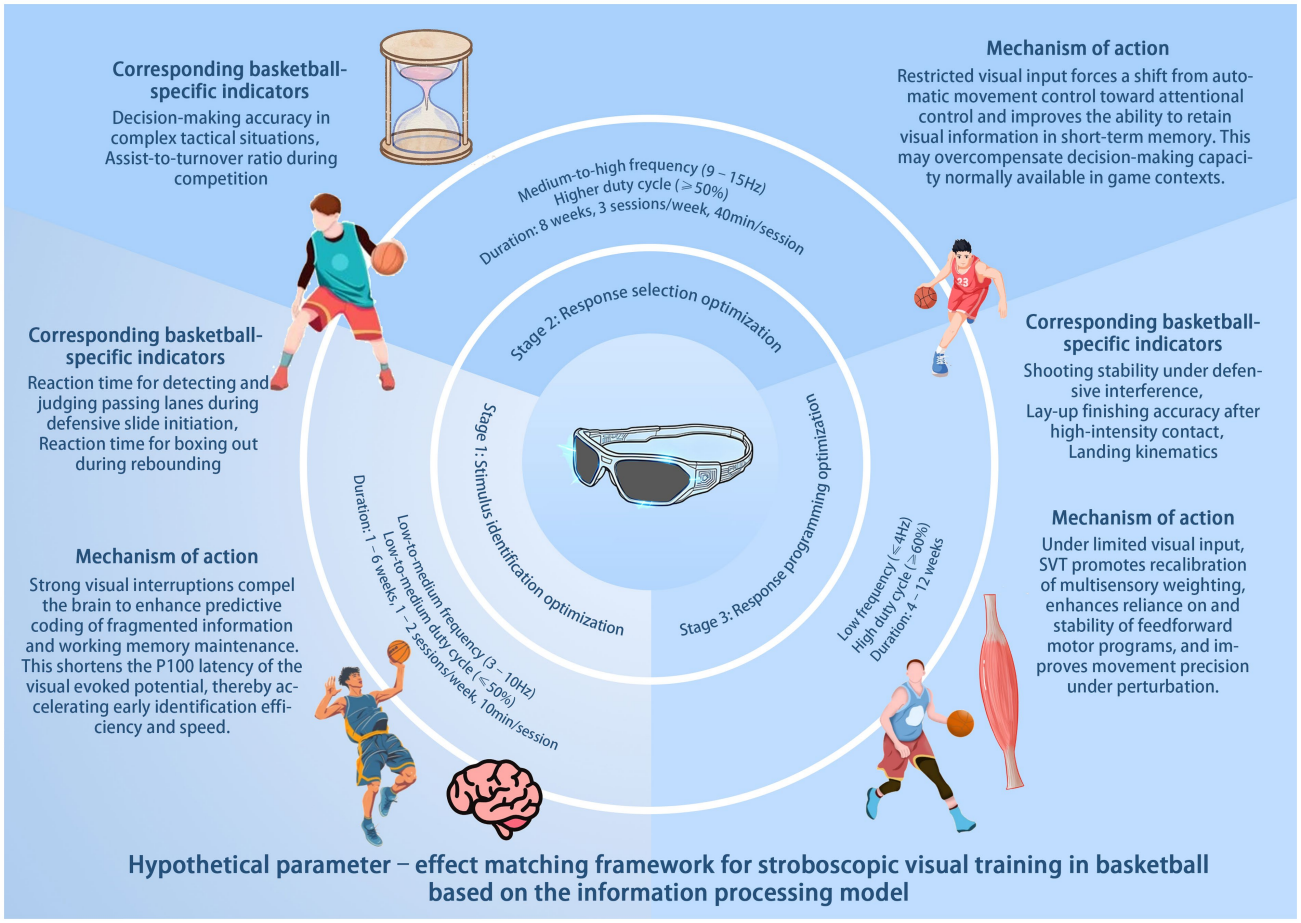
Hypothetical framework for SVT in basketball.

To provide guidance for training practice, we have proposed the hypothesis framework for SVT based on the information processing stages, derived from indirect evidence (see Figure 1). In Stage 1, reaction training incorporating SVT is conducted in a small-sided game simulation format (Zhang et al., 2025). Stage 2 builds on Stage 1 by incorporating the viewing of video-based simulated game environments for training (Pagé et al., 2019). Stage 3 employs plyometric training in the form of jumps and strength and balance training in the form of functional exercises under SVT-assisted conditions (Zhang et al., 2025).

Through indirect evidence, this hypothesis framework divides the potential benefits of SVT into three processing stages based on the information processing model and specifies the hypothesized parameters, neural mechanisms, and basketball-specific assessment indicators corresponding to each stage. This provides an evidence-based, referenceable hypothesis framework to address the questions of how to train and what to train with SVT in basketball.

## 8 Discussion

### 8.1 Neurophysiological and cognitive-psychological interpretation supporting the matching hypothesis framework

Existing research evidence provides a degree of support for the staged matching hypothesis framework proposed in this review. In stage one, which corresponds to training at a medium frequency and low-to-moderate duty cycle, the shortening of reaction time is accompanied by a shortened latency of the visual evoked potential P100 component, suggesting that SVT may accelerate early visual processing (Zwierko et al., 2024b). Although that study was conducted with handball players, P100 latency, as an objective indicator reflecting the conduction speed of the visual pathway, offers a possible neural explanation for the improvement of indicators such as defensive reaction speed in basketball. Additionally, while existing reviews have summarized the effects of SVT on visual cognition, direct evidence of P100 latency changes remains limited to a few studies (Wilkins et al., 2017).

Stage two may be influenced by the visual N2 potential, a component associated with the visuomotor system. Its latency reflects the speed of initiation and completion of cognitive control processes. SVT intervention has been shown to shorten N2 latency; a shorter latency implies faster detection of conflict or completion of response inhibition and decision evaluation, potentially influencing decision-making processing efficiency (Huelsduenker et al., 2021b). SVT intervention has also been accompanied by changes in short-term memory capacity. Open-skill sports like basketball place demands on short-term memory (Appelbaum et al., 2012). This suggests that an increase in short-term memory capacity may be associated with more efficient decision-making or higher decision-making accuracy.

Stage three is associated with the multisensory weight recalibration mechanism associated with SVT. Following SVT intervention, the nervous system’s ability to process and respond to unexpected sensory input is altered (Lu et al., 2025). This alteration may be mediated by a sensory reweighting mechanism, whereby the central nervous system reallocates the relative weights of visual, vestibular, and proprioceptive inputs in response to changing sensory conditions to maintain postural stability (Kim et al., 2017). SVT may influence this process by intermittently perturbing visual input, thereby increasing reliance on vestibular and proprioceptive feedback. Moreover, repeated exposure to such sensory perturbations may lead to changes within the central nervous system, particularly in brain regions involved in sensorimotor integration, such as the cerebellum, sensorimotor cortex, and superior parietal lobule (Nishimoto et al., 2023). These adaptive changes can indirectly influence motor planning and neuromuscular coordination, enabling postural responses to unpredictable stimuli.

Although the above associations remain inferential at present, prior SVT research provides indirect behavioral and neural evidence at multiple levels. At the behavioral level, during landing tasks under stroboscopic visual restriction, athletes exhibited longer stabilization times and a higher reactive strength index, which may stem from an increased reliance on and integration of proprioceptive feedback by the motor system to compensate for the lack of visual information. This may improve the response programming process—that is, more precise setting of movement parameters prior to execution—manifesting as motor control precision and efficiency (Kim et al., 2017; Grooms et al., 2018; Lu et al., 2025). At the cognitive level, research indicates that SVT can enhance athletes’ ability to anticipate dynamic events temporally (Wilkins and Appelbaum, 2019), which is consistent with the above speculation. At the neural level, increased time-frequency domain excitability may reflect the brain’s enhancement of intra- and inter-cortical functional connectivity and signal transmission efficiency to compensate for the unreliability of visual input. This can be understood as the neural basis underlying improved motor programming and sensorimotor integration (Yu et al., 2024). At the motor control level, relatively direct evidence shows that SVT can increase ankle dorsiflexion and eversion angles, and activate the tibialis anterior and peroneus longus muscles in patients with chronic ankle instability. This ankle preparation and muscle pre-activation reduces lower limb loading, enabling a more stable movement posture (Lee et al., 2024).

In summary, although current research on the neural mechanisms of SVT is based on indirect evidence or conducted in rehabilitation populations, findings from visual evoked potentials, event-related potentials, and sensorimotor integration studies show potential consistency with the three-phase framework proposed here, suggesting that SVT may improve information processing efficiency through multi-level neural plasticity. It should be noted that the above theoretical interpretations remain within the confines of this model and represent hypotheses rather than verified conclusions; future studies may test and verify these hypotheses.

### 8.2 Limitations of the review

Across the cross-sport evidence reviewed here, SVT offers multi-level support for the visual-cognitive demands of basketball, with potential benefits for attention, concentration, balance, reaction time, temporal anticipation, and decision-making in basketball players. The transferability of these benefits, however, remains to be verified in dedicated basketball-specific studies. Regarding evidence quality, the present narrative review has the following limitations: first, basketball-specific SVT studies are very limited, requiring substantial reliance on cross-sport evidence; second, the included studies are heterogeneous in design (randomized controlled trials, quasi-experimental studies, rehabilitation studies), sample characteristics (athlete level, sex ratio), and outcome measures; third, owing to the methodological constraints of narrative reviews, effect sizes from the original studies were not pooled, and the magnitude and consistency/variability of reported effects across meta-analyses were summarized qualitatively rather than statistically; and fourth, evidence for stage three (response programming) is predominantly drawn from the rehabilitation literature, and its extrapolation to healthy athletes warrants caution. Regarding the nature of the framework, the staged parameter–effect theoretical framework proposed here is a theoretically derived hypothesis based on the above evidence, rather than an empirically validated mature model; the parameter recommendations for each stage (e.g., frequency, duty cycle, training duration) represent trend-based syntheses of existing cross-sport research, and specific values await further validation through basketball-specific empirical research. Future studies should treat this framework as a starting point for hypothesis-driven research rather than as a definitive training guide; priority directions include randomized controlled trials with basketball players, neuroimaging-coupled investigations, and ecological-validity tests in real game contexts.

## References

[ref1] Aglioti S. M. Cesari P. Romani M. Urgesi C. (2008). Action anticipation and motor resonance in elite basketball players. Nat. Neurosci. 11, 1109–1116. doi: 10.1038/nn.2182, 19160510

[ref2] Alemanno M. Di Pompeo I. Marcaccio M. Canini D. Curcio G. Migliore S. (2025). From gaze to game: a systematic review of eye-tracking applications in basketball. Brain Sci. 15:421. doi: 10.3390/brainsci15040421, 40309899 PMC12025553

[ref3] Appelbaum L. G. Cain M. S. Schroeder J. E. Darling E. F. Mitroff S. R. (2012). Stroboscopic visual training improves information encoding in short-term memory. Atten. Percept. Psychophys. 74, 1681–1691. doi: 10.3758/s13414-012-0344-6, 22810559

[ref4] Appelbaum L. G. Erickson G. (2018). Sports vision training: a review of the state-of-the-art in digital training techniques. Int. Rev. Sport Exerc. Psychol. 11, 160–189. doi: 10.1080/1750984X.2016.1266376

[ref5] Appelbaum L. G. Schroeder J. E. Cain M. S. Mitroff S. R. (2011). Improved visual cognition through stroboscopic training. Front. Psychol. 2:276. doi: 10.3389/fpsyg.2011.00276, 22059078 PMC3203550

[ref6] Beavan A. Hanke L. Spielmann J. Skorski S. Mayer J. Meyer T. . (2021). The effect of stroboscopic vision on performance in a football specific assessment. Sci. Med. Footb. 5, 317–322. doi: 10.1080/24733938.2020.1862420, 35077302

[ref7] Czyż S. H. (2021). Variability of practice, information processing, and decision making-how much do we know? Front. Psychol. 12:639131. doi: 10.3389/fpsyg.2021.639131, 33679566 PMC7933225

[ref8] Das J. Walker R. Barry G. Vitório R. Stuart S. Morris R. (2023). Stroboscopic visual training: the potential for clinical application in neurological populations. PLOS Digit Health 2:e0000335. doi: 10.1371/journal.pdig.0000335, 37611053 PMC10446176

[ref9] Demir O. B. Bilgin A. (2025). The effect of balance training with stroboscopic glasses on postural stability and activity level in patients: a meta-analysis. Afr. Health Sci. 25, 184–195. doi: 10.4314/ahs.v25i3.23, 41179548 PMC12573648

[ref10] Ehmann P. Beavan A. Spielmann J. Ruf L. Mayer J. Rohrmann S. . (2021). 360°-multiple object tracking in team sport athletes: reliability and relationship to visuospatial cognitive functions. Psychol. Sport Exerc. 55:101952. doi: 10.1016/j.psychsport.2021.101952

[ref11] Ellison P. Jones C. Sparks S. A. Murphy P. N. Page R. M. Carnegie E. . (2020). The effect of stroboscopic visual training on eye–hand coordination. Sport Sci. Health 16, 401–410. doi: 10.1007/s11332-019-00615-4

[ref12] Fransen J. Lovell T. W. J. Bennett K. J. M. Deprez D. Deconinck F. J. A. Lenoir M. . (2017). The influence of restricted visual feedback on dribbling performance in youth soccer players. Mot. Control. 21, 158–167. doi: 10.1123/mc.2015-0059, 27111662

[ref13] Grooms D. Appelbaum G. Onate J. (2015). Neuroplasticity following anterior cruciate ligament injury: a framework for visual-motor training approaches in rehabilitation. J. Orthop. Sports Phys. Ther. 45, 381–393. doi: 10.2519/jospt.2015.5549, 25579692

[ref14] Grooms D. R. Chaudhari A. Page S. J. Nichols-Larsen D. S. Onate J. A. (2018). Visual-motor control of drop landing after anterior cruciate ligament reconstruction. J. Athl. Train. 53, 486–496. doi: 10.4085/1062-6050-178-16, 29749751 PMC6107770

[ref15] Güldenpenning I. Kunde W. Weigelt M. (2020). Cognitive load reduces interference by head fakes in basketball. Acta Psychol. 203:103013. doi: 10.1016/j.actpsy.2020.103013, 31955031

[ref16] Haberstroh T. (2016) How do Kawhi Leonard — and Steph curry — train their brains? Strobe lights (yes, really). Available online at: https://www.espn.com/nba/story/_/id/18002545/kawhi-leonard-strobe-light-training-nba. (Accessed March 15, 2026).

[ref17] Han S. Lee H. Son S. J. Hopkins J. T. (2022). The effects of visual feedback disruption on postural control with chronic ankle instability. J. Sci. Med. Sport 25, 53–57. doi: 10.1016/j.jsams.2021.07.014, 34393051

[ref18] Harris D. O’Malley C. Beck D. Zahno S. Arthur T. (2025) Predictive processing in sport and exercise science: a scoping review and theoretical overview 10.1186/s40798-026-01092-z PMC13481637 42606774

[ref19] Heilmann F. Weinberg H. Wollny R. (2022). The impact of practicing open- vs. closed-skill sports on executive functions-a meta-analytic and systematic review with a focus on characteristics of sports. Brain Sci. 12:1071. doi: 10.3390/brainsci12081071, 36009134 PMC9406193

[ref20] Huelsduenker T. Gunasekara N. Mierau A. (2021a). Short-and long-term stroboscopic training effects on Visuomotor performance in elite youth sports. Part 1: reaction and behavior. Med. Sci. Sports Exerc. 53, 960–972. doi: 10.1249/mss.0000000000002541, 33060548

[ref21] Huelsduenker T. Gunasekara N. Mierau A. (2021b). Short- and long-term stroboscopic training effects on visuomotor performance in elite youth sports. Part 2: brain–behavior mechanisms. Med. Sci. Sports Exerc. 53, 973–985. doi: 10.1249/mss.0000000000002543, 33060549

[ref22] Hülsdünker T. Rentz C. Ruhnow D. Käsbauer H. Strüder H. K. Mierau A. (2019). The effect of 4-week stroboscopic training on visual function and sport-specific Visuomotor performance in top-level badminton players. Int. J. Sports Physiol. Perform. 14, 343–350. doi: 10.1123/ijspp.2018-0302, 30160560

[ref23] Jin P. Ge Z. Fan T. (2023). Research on visual search behaviors of basketball players at different levels of sports expertise. Sci. Rep. 13:1406. doi: 10.1038/s41598-023-28754-2, 36697486 PMC9876905

[ref24] Jothi S. Dhakshinamoorthy J. Kothandaraman K. (2025). Effect of stroboscopic visual training in athletes. J. Hum. Sport Exerc. 20, 562–573. doi: 10.55860/vm1j3k88

[ref25] Kim K. M. Estudillo-Martínez M. D. Castellote-Caballero Y. Estepa-Gallego A. Cruz-Díaz D. (2021). Short-term effects of balance training with stroboscopic vision for patients with chronic ankle instability: a single-blinded randomized controlled trial. Int. J. Environ. Res. Public Health 18:5364. doi: 10.3390/ijerph18105364, 34069907 PMC8157596

[ref26] Kim K.-M. Kim J.-S. Grooms D. R. (2017). Stroboscopic vision to induce sensory reweighting during postural control. J. Sport Rehabil. 26, 1–11. doi: 10.1123/jsr.2017-0035, 28605310

[ref27] Kroll M. Preuss J. Ness B. M. Dolny M. Louder T. (2023). Effect of stroboscopic vision on depth jump performance in female NCAA division I volleyball athletes. Sports Biomech. 22, 1016–1026. doi: 10.1080/14763141.2020.1773917, 32510290

[ref28] Kunde W. Wühr P. (2006). Sequential modulations of correspondence effects across spatial dimensions and tasks. Mem. Cogn. 34, 356–367. doi: 10.3758/bf03193413, 16752599

[ref29] Lee H. Han S. Hopkins J. T. (2022a). Altered visual reliance induced by stroboscopic glasses during postural control. Int. J. Environ. Res. Public Health 19:2076. doi: 10.3390/ijerph19042076, 35206263 PMC8872389

[ref30] Lee H. Han S. Hopkins J. T. (2024). Balance training with stroboscopic glasses and Neuromechanics in patients with chronic ankle instability during a single-legged drop landing. J. Athl. Train. 59, 633–640. doi: 10.4085/1062-6050-0605.22, 37459365 PMC11220764

[ref31] Lee H. Han S. Page G. Bruening D. A. Seeley M. K. Hopkins J. T. (2022b). Effects of balance training with stroboscopic glasses on postural control in chronic ankle instability patients. Scand. J. Med. Sci. Sports 32, 576–587. doi: 10.1111/sms.14098, 34775656

[ref32] Li Y. Feng T. Zhang F. Asgher U. Yan B. Peng T. (2023). Visual search strategies of performance monitoring used in action anticipation of basketball players. Brain Behav. 13:e3298. doi: 10.1002/brb3.3298, 37872861 PMC10726756

[ref33] Li Y. Li S. Yang J. Hao Y. (2026). The effects of stroboscopic visual training on coordination, change-of-direction, and decision-making performance in collegiate basketball players. Front. Psychol. 17:1750065. doi: 10.3389/fpsyg.2026.1750065, 41788264 PMC12956791

[ref34] Li T. Wang X. Wu Z. Liang Y. (2024). The effect of stroboscopic vision training on the performance of elite curling athletes. Sci. Rep. 14:31730. doi: 10.1038/s41598-024-82685-0, 39738499 PMC11685645

[ref35] Lochhead L. Feng J. Laby D. M. Appelbaum L. G. (2024). Training vision in athletes to improve sports performance: a systematic review of the literature. Int. Rev. Sport Exerc. Psychol., 1–23. doi: 10.1080/1750984x.2024.2437385, 37339054

[ref36] Lu M. Zhai Y. Peng H. Cao J. Yang Y. Chen L. (2025). Effects of balance training combined with stroboscopic visual training on balance ability in college-aged male soccer players. BMC Sports Sci. Med. Rehabil. 17:207. doi: 10.1186/s13102-025-01255-7, 40682165 PMC12272995

[ref37] Lucia S. Bianco V. Boccacci L. Di Russo F. (2021). Effects of a cognitive-motor training on anticipatory brain functions and sport performance in semi-elite basketball players. Brain Sci. 12:68. doi: 10.3390/brainsci12010068, 35053809 PMC8773627

[ref38] Luo Y. Cao Y. Pan X. Li S. Koh D. Shi Y. (2025a). Effects of stroboscopic visual training on reaction time and movement accuracy in collegiate athletes: a systematic review and meta-analysis. Sci. Rep. 15:25151. doi: 10.1038/s41598-025-10393-4, 40646137 PMC12254360

[ref39] Luo Y. Zhang X. Li S. Cao Y. Pan X. Koh D. (2025b). Stroboscopic visual training combined with balance exercises for chronic ankle instability: a systematic review and meta-analysis. J. Orthop. Surg. Res. 21:44. doi: 10.1186/s13018-025-06353-3, 41413570 PMC12829179

[ref40] Mangine G. T. Hoffman J. R. Wells A. J. Gonzalez A. M. Rogowski J. P. Townsend J. R. . (2014). Visual tracking speed is related to basketball-specific measures of performance in NBA players. J. Strength Cond. Res. 28, 2406–2414. doi: 10.1519/jsc.0000000000000550, 24875429

[ref41] Mann D. L. Abernethy B. Farrow D. (2010). Visual information underpinning skilled anticipation: the effect of blur on a coupled and uncoupled in situ anticipatory response. Atten. Percept. Psychophys. 72, 1317–1326. doi: 10.3758/app.72.5.1317, 20601713

[ref42] Martín A. Sfer A. M. D'Urso Villar M. A. Barraza J. F. (2017). Position affects performance in multiple-object tracking in Rugby union players. Front. Psychol. 8:1494. doi: 10.3389/fpsyg.2017.01494, 28951725 PMC5599788

[ref43] Michaels C. (2000). Information, perception, and action: what should ecological psychologists learn from Milner and Goodale (1995)? Ecol Psychol 12, 241–258. doi: 10.1207/S15326969ECO1203_4

[ref44] Mitroff S. R. Friesen P. Bennett D. Yoo H. Reichow A. W. (2013). Enhancing ice hockey skills through stroboscopic visual training: a pilot study. Athl. Train. Sports Health Care 5, 261–264. doi: 10.3928/19425864-20131030-02

[ref45] Moeinirad S. Abdoli B. Farsi A. Ahmadi N. (2022). Training visual attention improves basketball three-point shot performance under pressure. Sport Sci. Health 18, 853–861. doi: 10.1007/s11332-021-00866-0

[ref46] Moore L. J. Vine S. J. Cooke A. Ring C. Wilson M. R. (2012). Quiet eye training expedites motor learning and aids performance under heightened anxiety: the roles of response programming and external attention. Psychophysiology 49, 1005–1015. doi: 10.1111/j.1469-8986.2012.01379.x, 22564009

[ref47] Nishimoto R. Fujiwara S. Kutoku Y. Ogata T. Mihara M. (2023). Effect of dual-task interaction combining postural and visual perturbations on cortical activity and postural control ability. NeuroImage 280:120352. doi: 10.1016/j.neuroimage.2023.120352, 37648121

[ref48] Pagé C. Bernier P.-M. Trempe M. (2019). Using video simulations and virtual reality to improve decision-making skills in basketball. J. Sports Sci. 37, 2403–2410. doi: 10.1080/02640414.2019.1638193, 31280685

[ref49] Raichin A. Shkedy Rabani A. Shmuelof L. (2021). Motor skill training without online visual feedback enhances feedforward control. J. Neurophysiol. 126, 1604–1613. doi: 10.1152/jn.00145.2021, 34525324

[ref50] Ren Y. Zhang J. Sun X. Qi F. Guo F. (2025). The effects of stroboscopic visual training on human cognitive function and motor performance: a systematic review. Front. Physiol. 16:1708783. doi: 10.3389/fphys.2025.1708783, 41394926 PMC12695576

[ref51] Romeas T. Guldner A. Faubert J. (2016). 3D-multiple object tracking training task improves passing decision-making accuracy in soccer players. Psychol. Sport Exerc. 22, 1–9. doi: 10.1016/j.psychsport.2015.06.002

[ref52] Ryu D. Abernethy B. Mann D. L. Poolton J. M. (2015). The contributions of central and peripheral vision to expertise in basketball: how blur helps to provide a clearer picture. J. Exp. Psychol. Hum. Percept. Perform. 41, 167–185. doi: 10.1037/a0038306, 25485663

[ref53] Schmidt R. A. Lee T. D. Winstein C. Wulf G. Zelaznik H. N. (2018). Motor Control and Learning: A Behavioral Emphasis. 6th Edn Champaign: Human Kinetics.

[ref54] Smith T. Q. Mitroff S. R. (2012). Stroboscopic training enhances anticipatory timing. Int. J. Exerc. Sci. 5, 344–353. doi: 10.70252/otsw1297, 27182391 PMC4738880

[ref55] Uchida Y. Mizuguchi N. Honda M. Kanosue K. (2014). Prediction of shot success for basketball free throws: visual search strategy. Eur. J. Sport Sci. 14, 426–432. doi: 10.1080/17461391.2013.866166, 24319995

[ref56] Uzlaşır S. Özdıraz K. Y. Dağ O. Tunay V. B. (2021). The effects of stroboscopic balance training on cortical activities in athletes with chronic ankle instability. Phys. Ther. Sport 50, 50–58. doi: 10.1016/j.ptsp.2021.03.014, 33865218

[ref57] Vickers J. N. Causer J. Vanhooren D. (2019). The role of quiet eye timing and location in the basketball three-point shot: a new research paradigm. Front. Psychol. 10:2424. doi: 10.3389/fpsyg.2019.02424, 31736825 PMC6836760

[ref58] Vine S. J. Wilson M. R. (2011). The influence of quiet eye training and pressure on attention and visuo-motor control. Acta Psychol. 136, 340–346. doi: 10.1016/j.actpsy.2010.12.008, 21276584

[ref59] Wang R. Li S. Wu Y. Liu H. Zhang Q. (2025). Effects of stroboscopic visual training on reaction time and decision-making ability in athletes: a systematic review and meta-analysis. Front. Psychol. 16:1697425. doi: 10.3389/fpsyg.2025.1697425, 41333302 PMC12665768

[ref60] Weigelt M. Güldenpenning I. Steggemann-Weinrich Y. Alhaj Ahmad Alaboud M. Kunde W. (2017). Control over the processing of the opponent's gaze direction in basketball experts. Psychon. Bull. Rev. 24, 828–834. doi: 10.3758/s13423-016-1140-4, 27542803

[ref61] Wilkins L. Appelbaum L. G. (2019). An early review of stroboscopic visual training: insights, challenges and accomplishments to guide future studies. Int. Rev. Sport Exerc. Psychol. 13, 65–80. doi: 10.1080/1750984x.2019.1582081

[ref62] Wilkins L. Nelson C. Tweddle S. (2017). Stroboscopic visual training: a pilot study with three elite youth football goalkeepers. J. Cogn. Enhanc. 2, 3–11. doi: 10.1007/s41465-017-0038-z, 30311153

[ref63] Wohl T. R. Criss C. R. Haggerty A. L. Rush J. L. Simon J. E. Grooms D. R. (2024). The impact of visual perturbation neuromuscular training on landing mechanics and neural activity: a pilot study. Int. J. Sports Phys. Ther. 19, 1333–1347. doi: 10.26603/001c.123958, 39502544 PMC11534169

[ref64] Wu Y. Zeng Y. Zhang L. Wang S. Wang D. Tan X. . (2013). The role of visual perception in action anticipation in basketball athletes. Neuroscience 237, 29–41. doi: 10.1016/j.neuroscience.2013.01.048, 23384606

[ref65] Yu B. You Y. Li Y. Chen J. Zhou H. Wang J. . (2024). Effects of intermittent visual feedback on EEG characteristics during motor preparation and execution in a goal-directed task. Front. Hum. Neurosci. 18:1371476. doi: 10.3389/fnhum.2024.1371476, 39726693 PMC11669603

[ref66] Yu H. Zhu Y. Gai B. (2025). Complexity amplifies expertise: visual search and anticipatory skill adaptations in skilled basketball players. Percept. Mot. Skills. doi: 10.1177/00315125251394789, 41165080

[ref67] Zemková E. Horníková H. Skala F. Argaj G. (2025). Association of Game-Specific Performance of young skilled basketball players with sensorimotor factors of agility skills. J. Hum. Kinet. 96, 213–223. doi: 10.5114/jhk/202260, 40453889 PMC12121887

[ref68] Zhang M. Li F. Jiao J. Liang W. Gomez M. A. Scanlan A. T. (2025). Effects of different training methods on open-skill and closed-skill agility in basketball players: a systematic review and meta-analysis. Sports Med. Open 11:50. doi: 10.1186/s40798-025-00842-9, 40332702 PMC12058619

[ref69] Zwierko T. Jedziniak W. Domaradzki J. Zwierko M. Opolska M. Lubiński W. (2024b). Electrophysiological evidence of stroboscopic training in elite handball players: visual evoked potentials study. J. Hum. Kinet. 90, 57–69. doi: 10.5114/jhk/169443, 38380298 PMC10875695

[ref70] Zwierko M. Jedziniak W. Popowczak M. Rokita A. (2023). Effects of in-situ stroboscopic training on visual, visuomotor and reactive agility in youth volleyball players. PeerJ 11:e15213. doi: 10.7717/peerj.15213, 37250711 PMC10211363

[ref71] Zwierko M. Jedziniak W. Popowczak M. Rokita A. (2024a). Effects of six-week stroboscopic training program on visuomotor reaction speed in goal-directed movements in young volleyball players: a study focusing on agility performance. BMC Sports Sci. Med. Rehabil. 16:59. doi: 10.1186/s13102-024-00848-y, 38424539 PMC10905827

